# Orthorexia nervosa: Why research based on imperfect measures may still be useful

**DOI:** 10.7189/jogh.14.03053

**Published:** 2024-12-06

**Authors:** José Francisco López-Gil, Pedro Juan Tárraga-López, Maria S Hershey, Rubén López-Bueno, Héctor Gutiérrez-Espinoza, Antonio Soler-Marín, Alejandro Fernández-Montero, Desirée Victoria-Montesinos

**Affiliations:** 1 One Health Research Group, Universidad de Las Américas, Quito, Ecuador; 2 Departamento de Ciencias Médicas, Facultad de Medicina, Universidad Castilla-La Mancha, Albacete, Spain; 3 Department of Environmental Health, Harvard University T.H. Chan School of Public Health, Boston, Massachusetts, USA; 4 University of Navarra, Department of Preventive Medicine and Public Health, School of Medicine, Pamplona, Spain; 5 Department of Physical Medicine and Nursing, University of Zaragoza, Zaragoza, Spain; 6 Faculty of Education, Universidad Autónoma de Chile, Chile; 7 Faculty of Pharmacy and Nutrition, Universidad Católica San Antonio de Murcia, Murcia, Spain; 8 Department of Occupational Medicine, University of Navarra, Instituto de Investigación Sanitaria de Navarra, Pamplona, Spain

The purpose of this commentary is to respond to the critiques raised in the viewpoint titled ‘Orthorexia nervosa: Research based on invalid measures is invalid’ by Barrada and Meule [1]. We acknowledge the importance of rigorous psychometric evaluation and the concerns raised regarding the use of the ORTO-15 questionnaire in our systematic review and meta-analysis, ‘Overall proportion of orthorexia nervosa symptoms: A systematic review and meta-analysis including 30 476 individuals from 18 countries’ [2]. We respect their expertise and understand their concerns about the psychometric properties of the ORTO-15 questionnaire [3]. However, we believe it is necessary to clarify and address several points raised in their letter.

First and foremost, we agree with Barrada and Meule that the ORTO-15 questionnaire has known psychometric limitations, including issues with reliability and validity. These limitations are indeed significant and warrant careful consideration when interpreting the results derived from studies using this tool. We acknowledged some of these limitations in the discussion section of our paper, where we noted the need for improved psychometric instruments in the field of orthorexia nervosa (ON) research. In addition, we concur with the recommendation to move towards more theoretically sound and psychometrically valid tools for future studies assessing ON. We support Barrada and Meule’s call for abandoning the use of the ORTO-15 in favour of more psychometrically sound measures. Therefore, our study should be seen as a call to action for the research community to address these measurement issues urgently.

The primary aim of our study was to provide an overview of the prevalence of ON symptoms using the most commonly employed tool in the literature, the ORTO-15. It did not aim to determine the psychometric properties of the tool. Our intent was to synthesise available data and highlight the widespread use of this tool, while also pointing out the high prevalence rates observed and the potential public health implications. This approach is common in meta-analytic studies, which often rely on the measures used in the primary studies, despite potential limitations. The methodology of our work and the statistical analyses are adequate and sophisticated, which is what depends on us (and something that Barrada and Meule have not objected to). Therefore, we believe that our study can serve as a critical stepping stone that underscores the urgent need for more accurate diagnostic tools and standardised criteria for ON, not to claim definitive prevalence rates.

However, we also recognise the critique of using a categorical approach to define ON based on arbitrary cut-off points. As the field evolves, a dimensional approach to understanding ON symptoms, considering the severity and impact on daily functioning, may indeed be more appropriate. However, in this systematic review and meta-analysis we used the cut-off points commonly applied in the literature. Therefore, our findings of approximately 27.5% prevalence, based on the current cut-off of <35 points on the ORTO-15, should be interpreted with caution and in the context of ongoing debates about the best methods to assess ON.

Regarding the concern that our findings may contribute to the pathologisation of healthy eating behaviours, we want to clarify that our intention is not to pathologise, but to highlight the potential public health implications of an excessive preoccupation with healthy eating. As noted in our paper, orthorexia nervosa is characterised by significant distress or impairment in important areas of functioning due to an obsession with healthy eating. This distinction is crucial to avoid mislabelling those who are simply health-conscious. Additionally, we limit ourselves to reporting the synthesis of the data, without making value judgments or trying to magnify the problem (ie, pathologization of healthy eating behaviours).

While Barrada and Meule’s critique is valuable, it is important to recognise the contributions that studies like ours make to the field. By compiling and analysing data from multiple studies, we help to identify patterns and areas of concern that might otherwise go unnoticed. Our findings, despite being based on a tool with limitations, highlight the potential magnitude of the number of studies assessing ON (using the ORTO-15) and call attention to the necessity for improved research methodologies.

Finally, we believe that, while critical evaluation is essential, it is equally important to present a balanced perspective that recognizes both the limitations and contributions of existing research. Titles such as ‘Research based on invalid measures is invalid’ can be perceived as dismissive of efforts to advance understanding in a complex and evolving field. Furthermore, this type of title does not do justice to the objective and contributions of our work. Describing the entirety of research utilising the ORTO-15 as ‘noise’ and likening it to ‘garbage’ can be perceived as dismissive and does not contribute to constructive scientific discourse. While we understand the importance of rigour, we believe that such sweeping generalisations do not adequately acknowledge the nuanced contributions of existing research to the field of ON. Hence, we advocate for a more constructive approach that encourages improvement without undermining the progress made thus far.

Additionally, while we recognise the importance of letters to the editor in stimulating scholarly dialogue, we believe that comprehensive literature reviews and meta-analyses also play a crucial role in advancing scientific knowledge. It remains to be seen whether a thorough review of the literature and an accompanying meta-analysis provide more scientific value than writing letters to the editor, and we will not be the ones to make that determination. Ultimately, it is not for us to determine which approach contributes more significantly to the field; maybe both have their respective merits.

We appreciate the dialogue initiated by Barrada and Meule and hope that it stimulates further research and development of better diagnostic tools for ON. We are committed to contributing to the advancement of this field and welcome continued discussions on how best to achieve this goal.
